# Evaluation of Adhesive Characteristics of *L. plantarum* and *L. reuteri* Isolated from Weaned Piglets

**DOI:** 10.3390/microorganisms9081587

**Published:** 2021-07-26

**Authors:** Matteo Dell’Anno, Carlotta Giromini, Serena Reggi, Mariagrazia Cavalleri, Alessandra Moscatelli, Elisabetta Onelli, Raffaella Rebucci, Tamil Selvi Sundaram, Simona Coranelli, Ambra Spalletta, Antonella Baldi, Luciana Rossi

**Affiliations:** 1 Department of Health, Animal Science and Food Safety “Carlo Cantoni” (VESPA), Università Degli Studi di Milano, 26900 Lodi, Italy; Matteo.DellAnno@unimi.it (M.D.); serena.reggi@unimi.it (S.R.); mariagrazia.cavalleri@studenti.unimi.it (M.C.); raffaella.rebucci@unimi.it (R.R.); tamil.sundaram@unimi.it (T.S.S.); antonella.baldi@unimi.it (A.B.); luciana.rossi@unimi.it (L.R.); 2 Department of Biosciences, Università Degli Studi di Milano, 20133 Milan, Italy; alessandra.moscatelli@unimi.it (A.M.); elisabetta.onelli@unimi.it (E.O.); 3 Biotecnologie B.T. Srl, Todi, 06059 Perugia, Italy; scoranelli@biotecnologiebt.it (S.C.); aspalletta@biotecnologiebt.it (A.S.)

**Keywords:** probiotic, surface proteins, intestinal adhesion, gastro-intestinal system, *E. coli*, F18, pig, *Lactobacillus plantarum*, *Lactobacillus reuteri*, IPEC-J2

## Abstract

*Limosilactobacillus reuteri* and *Lactiplantibacillus plantarum* strains, previously isolated from weaned piglets, were considered for the evaluation of their adhesive characteristics. Lactobacilli were treated with LiCl in order to remove the surface protein layer, and probiotic activity was compared with those of untreated strains. The autoaggregation, co-aggregation to *E. coli* F18+, and adhesive abilities of LiCl-treated *Limosilactobacillus reuteri* and *Lactiplantibacillus plantarum* were significantly inhibited (*p* < 0.05) compared with the respective untreated strain. The hydrophobic and basic phenotypes were observed due to the strong affinity to chloroform and low adherence to ethyl acetate. In particular, *L. plantarum* showed higher hydrophobicity compared to *L. reuteri*, which may reflect their different colonizing ability. After treatment with LiCl to remove surface proteins, the adherence capabilities of *L. reuteri* and *L. casei* on IPEC-J2 cells decreased significantly (*p* < 0.001) and *L. reuteri* adhered more frequently. Sodium dodecyl sulphate-polyacrylamide gel electrophoresis (SDS-PAGE) showed that both *L. reuteri* and *L. plantarum* had several bands ranging from 20 to 100 kDa. Two-dimensional gel electrophoresis showed an acidic profile of the surface-layer polypeptides for both bacterial strains, and more studies are needed to characterize their profile and functions. The results confirm the pivotal role of surface proteins in the probiotic potential of *L. reuteri* and *L. plantarum*.

## 1. Introduction

Lactobacilli are part of the common flora in the porcine digestive tract [1]. Niu et al. [2] showed that the *Lactobacillus* genus accounts for approximately 15% of 16S rRNA in intestinal pig samples, regardless of age. Notably, while the swine faecal microbiota changed significantly across growth stages, the populations of lactobacilli remain almost stable.

Although several studies have shown the positive impact of *Limosilactobacillus reuteri* and *Lactiplantibacillus plantarum*, previously known as the *Lactobacillus* genus, on piglet’s performance improvement, diarrhoea prevention, stress alleviation, immunity, microbiota modulation, and carcass quality [3,4], they are not listed in the European register of additives for pigs. Moreover, the species- and strain-specific characteristics of lactobacilli that confer probiotic benefits are still not well-documented. Indeed, *L. plantarum* can be found in the European Register of Feed Additives according to European Regulation [5], under the categories of preservatives, silage additives, microorganisms, and gut flora stabilizers for fattening chickens. On the other hand, *L. reuteri* is mostly used as a probiotic in humans. Lactobacilli are generally selected as potential probiotics due to their natural ability to survive the digestive process. After the arrival in the intestinal section, lactobacilli can protect the host from pathogens by means of different mechanisms (competitive exclusion, bacteriocins production and stimulation of mucosal immunity) [6,7]. The adhesive capacity of bacteria to epithelial cells is one of the main probiotic characteristics [8]. The lactobacilli are Gram-positive bacteria characterized by a cell envelope consisting of a cell membrane and cell wall. These two layers are covered by several surface proteins, with the main function being as a protective sheath against environmental challenges. It has been previously proposed that bacteria surface proteins of lactobacilli are involved in cell protection and surface recognition and that they could be potential mediators of bacteria autoaggregation and adhesion to intestinal cells [9].

*L. reuteri* and *L. plantarum* were previously demonstrated to have a significant role in controlling diarrhoea in piglets [10]. In particular, dietary supplementation of 2 × 10^8^ CFU/g of *L. plantarum* and *L. reuteri* significantly reduced diarrhoea occurrence and had the lowest faecal score in our trial. *L. plantarum* showed the lowest diarrhoea frequency compared to the other bacterial strains and their combinations. *L. plantarum* and *L. reuteri* supplementation did not influence animal performance, total faecal bacteria, faecal lactobacilli and coliform. In addition, *L. plantarum* and *L. reuteri* showed high resistance to a wide range of pH and digestive processes [10]. In our attempt to study the adhesive characteristics in the probiotic activity of *L. reuteri* and *L. plantarum*, we investigated *Limosilactobacillus reuteri* and *Lactiplantibacillus plantarum* autoaggregation, co-aggregation capacity to *Escherichia coli* F18+, bacterial hydrophobicity, adhesion to swine intestinal IPEC-J2 cells and cell surface proteins characteristics.

## 2. Materials and Methods

### 2.1. Bacterial Strains and Culturing Conditions

Single colonies of *L. plantarum* and *L. reuteri*, isolated from swine, were obtained from the Biotecnologie B.T. (Perugia, Italy) strain collection [10] and grown in MRS agar medium. *L. plantarum* and *L. reuteri* strains were individually inoculated from our laboratory stock at −80 °C into the De Man, Rogosa and Sharpe (MRS) medium (Liofilchem, Italy) and incubated at 35 °C for 24 h under a microaerophilic atmosphere by adding sterile oil above the culture media.

### 2.2. Aggregative Abilities of LAB Strains

Lactobacilli were cultured in MRS broth at 35 °C for 24 h under a microaerophilic atmosphere by adding sterile oil above the culture media. Bacterial pellets were harvested by centrifugation (3500 rpm, 10 min), and cells were rinsed with PBS (1X) in order to reach an optical density (OD) of 0.2–0.3 at 600 nm wavelength using a spectrophotometer (Jasco V630 UV-VIS, JASCO Deutschland GmbH, Pfungstadt, Germany). Cell suspensions were vortexed for 6 s and incubated 6 h at room temperature. On millilitre of the upper part of the suspension was measured at 600 nm each hour. The percentage of auto-aggregation was then calculated.

$$\%\;autoaggregation=\left[1-\left(\frac{\mathrm{At}}{A0}\right)\right]\times 100$$

where At is the absorbance at different time points and A0 the initial one.

For co-aggregation abilities, resuspended LAB strains were mixed with aliquots of F18+ *E. coli* previously characterized for the presence of F18 adhesive fimbriae [11]. The *E. coli* culture was obtained by incubating the bacterial strain at 37 °C in Luria–Bertani medium under aerophilic conditions, and to left stir overnight at 110 rpm. Samples were mixed thoroughly for 10 s and incubated for 6 h at room temperature. One millilitre of the upper part of the suspension was measured at 600 nm each hour. The co-aggregation percentage was then calculated.

$$\%\;\mathrm{coaggregation}=\;\left\{\;\frac{\left[\left(\frac{\mathrm{Ax}+\mathrm{Ay}}{2}\right)-A\;\left(x+y\right)\right]}{\mathrm{Ax}+\mathrm{Ay}/2}\;\right\}\;\times 100$$

Ax and Ay indicate the individual proprieties of lactobacilli and *E. coli*, and A(x+y) express the combined aggregation of *L. plantarum* or *L. reuteri* and *E. coli*. The whole analyses were performed in two independent experiments including three replicates.

### 2.3. Determination of Bacterial Hydrophobicity

Microbial adhesion to solvents (MATS) was assessed according to Kos et al. [12]. Briefly, bacteria were harvested from the stationary phase after centrifugation (5000× *g* for 15 min). Samples were washed twice and resuspended in PBS (1X, pH 7.0) and brought to OD 0.6 at 600 nm. For the assay, 1 mL of the solvent was added to 3 mL of the bacterial suspension. After 10 min as preincubation at room temperature, samples were mixed for 2 min. After 20 min, the aqueous phase was removed to measure the OD at 600 nm (A1). The percentage of bacterial surface hydrophobicity was then calculated.

$$\left(\frac{\mathrm{Absorbance}\;\mathrm{before}\;\mathrm{mixing}-\mathrm{Absorbance}\;\mathrm{after}\;\mathrm{mixing}}{\mathrm{Absorbance}\;\mathrm{before}\;\mathrm{mixing}}\right)\times 100$$

For the evaluation of surface hydrophobicity, three solvents were tested: toluene (Titolchimica, Italy) as the apolar solvent; chloroform (Merck, Darmastadt, Germany) as monopolar basic and acidic solvent; and ethyl acetate (Carlo Erba Reagents S.A.S, Milan, Italy) as monopolar basic solvent [13]. The whole analyses were performed in two independent experiments including three replicates.

### 2.4. Cell Line and Culture Conditions

IPEC-J2 is a non-transformed cell line, derived from the jejunum epithelium of unsuckled piglets (DSMZ, Braunschweig, Germany). Cells used for the experiment were defrosted from a cryopreserved stock and cell passages of 24–28 were used for the experiments. Cells were routinely grown in a total volume of 100 mL of 1:1 of Dulbecco’s modified Eagle’s medium with stable L-glutamate and Ham’s F-12 mixture (DMEM/F12) (Immunological sciences, Società Italiana Chimici, Rome, Italy), plus 15 mM of HEPES (Sigma-Aldrich, Milano, Italy), 5% heat-inactivated foetal bovine serum (FBS) (Immunological sciences, Società Italiana Chimici, Rome, Italy), 1% penicillin (100 U/mL)/streptomycin (100 mg/mL) (Euroclone, Milano, Italy) and 1% GlutaMAX at 37 °C in a 5% CO_2_ atmosphere and subcultivated at 80% confluence. For adhesion assay, IPEC-J2 monolayers were prepared in a 2-well chamber slides system coated with collagen. After collagen coating, the cells were seeded at a concentration of 4 × 10^5^ cells/chamber to reach 80% confluence before lactobacilli addition.

### 2.5. Bacterial Adhesion Assay

Two-well chamber slides were used to study the adhesion ability of *L. plantarum*, *L. reuteri* and *L. casei* ATCC 393 (reference strain) to IPEC-J2 cells. Two millilitres of 48 h cultures of *L. plantarum*, *L. reuteri* and *L. casei* grown in 30 mL MRS broth (adjusted to 2.3 × 10^8^ CFU/mL) were centrifuged, washed, and resuspended in DMEM/F12 medium. Before the experiments, the cell medium was removed and 1 mL of each bacterial suspension was added to one well of each chamber slide, while the other well was filled with 1 mL of each bacterial suspension after LiCl (5 M) treatment [14]. Briefly, the cell pellet was resuspended in LiCl (5 M) and incubated at room temperature for 30 min. Bacteria were centrifugated at 5000× *g* for 15 min, and the cell pellet was washed twice with sterile saline solution before being resuspended in the cell medium. After 1 h incubation in a 5% CO_2_ atmosphere, the chamber slides were washed twice with PBS (1X) with Ca and Mg, and a 15 min fixation step with 500 µL of MetOH was made. Then, GIEMSA staining was performed with the GIEMSA dye diluted at a ratio of 1:20 with PBS (1X) for 30 min. After washing with deionized water, the glasses were left to dry overnight. Cells and bacteria in a 20 microscopy field (100× magnification with oil immersion) were randomly counted; the bacteria adherent to at least 200 cells were counted. The analyses were performed in three independent experiments including at least two technical replicates per treatment. The bacterial adherence value was defined as the number of the adhered bacteria per cell [15]. Data are expressed as adherent bacteria/number of cells.

### 2.6. Isolation and One and Two-Dimensional Gel Electrophoresis of S-Layer Proteins from L. plantarum and L. reuteri

S-layer proteins of lactobacilli were extracted by 5 M LiCl according to the method reported by Singh et al. [16]. Briefly, lactobacilli were incubated in 30 mL MRS at 35 °C in anaerobiosis conditions. Cells were collected and washed twice with sterile water. The pellet was treated with 5 M LiCl at 4 °C for 30 min. The supernatant was collected and dialyzed with PBS (1X) and concentrated. The extracted surface proteins were quantified using the Bradford method, with bovine serum albumin (BSA) as the standard [17]. For determination of the apparent molecular mass, SDS-PAGE was performed using a 10% (*w*/*v*) acrylamide gel in denaturing and reducing conditions. The gel was stained by Coomassie brilliant blue R-250 (Sigma, Saint Louis, MO, USA) and the intensity of bands was evaluated using ImageJ software. For two-dimensional gel electrophoresis, 25 µg of extracted S-layer proteins were precipitated with cold ethanol (1:5 *v*/*v*) and incubated for 30 min in ice. The pellet was obtained by centrifugation at 4 °C for 36 min at 20,627 *g* (15,000 rpm) in an ALC A21-C rotor. Ethanol was removed and the pellet was resuspended with 2D 200 µL of rehydration buffer (8 M urea, 4% CHAPS, 65 mM dithioerythritol, 0.5% bromophenol blue) supplemented with 2% 3–10 IPG buffer and loaded onto 7-cm non-linear pH 3–10 strips by overnight passive rehydration at room temperature. IEF was performed with Multifor II system (GE Healthcare, Chicago, IL, USA) according to Moscatelli et al. [18] (200 V for 1 h, 2000 V for 3 h and 3000 V for 3 h and 30 min). Focused strips were equilibrated in Buffer I (0.5 M TrisHCl, pH 6.8, 2% SDS, 6 M urea, 30% glycerol, 2% DTE) for 12 min and then for another 5 min in Buffer II (composition the same as Buffer I, but with 2.5% iodoacetamide instead of DTE) at room temperature. SDS-PAGE was run in 10% polyacrylamide gel (MiniVe Vertical Electrophoresis System, GE Healthcare, USA), as described previously [19]. The gels were silver-stained [20].

### 2.7. Statistical Analysis

The statistical analyses were performed through the software GraphPad Prism 9.0.1. The results of the aggregation, co-aggregation, cell surface hydrophobicity and adhesion assay for both the species were analysed through a one-way analysis of variance (ANOVA) procedure. Pairwise comparisons were evaluated using Tukey’s HSD test. Differences were considered statistically significant at *p* ≤ 0.05. Data were expressed as the least squares (LS) means ± standard error of mean (SEM).

## 3. Results

### 3.1. Aggregation Abilities

**Figure 1:**
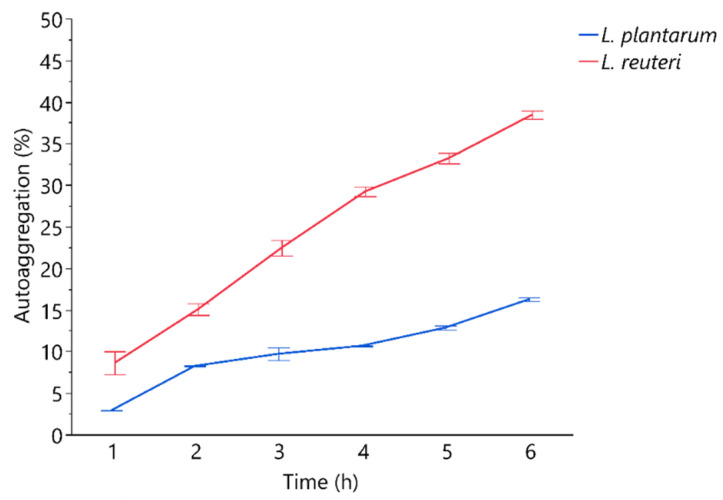
Percentages of autoaggregation measured at hourly bases. Data are presented as means ± standard deviations.

**Figure 2:**
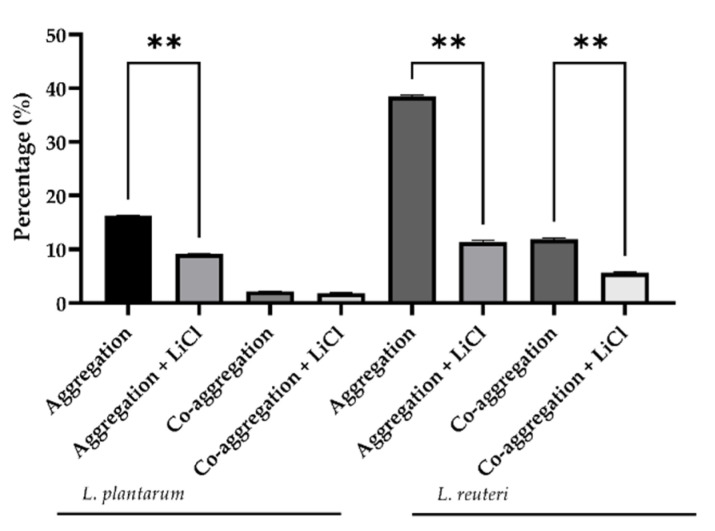
Percentage of *L. reuteri* and *L. plantarum* aggregation and co-aggregation with *E. coli* F18 measured at 6 h. Data are presented as LS mean ± SEM. ** indicates a statistically significant difference (*p* ≤ 0.001).

**Figure 3:**
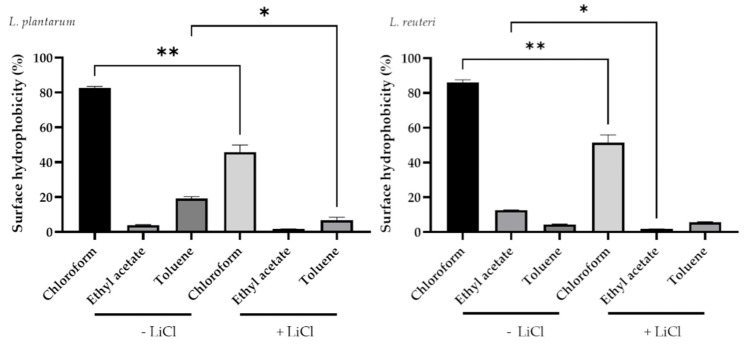
Effect of LiCl treatment on cell surface hydrophobicity of lactobacilli strains. Data are presented as LS means ± SEM. * Indicates a statistically significant difference (*p* ≤ 0.05); ** indicates a statistically significant difference (*p* ≤ 0.001).

The auto-and co-aggregation abilities of the *L. plantarum* and *L. reuteri* are summarized in Figure 1 and Figure 2. After 6 h of incubation, the highest percentages of aggregation were observed for *L. reuteri*. Both strains demonstrated co-aggregation ability with intestinal pathogen tested (*E. coli* F18+). The maximum autoaggregation and co-aggregation were shown by *L* *reuteri* at 6 h (38.46 ± 0.49% and 11.60 ± 0.30%, respectively). The auto-aggregation and co-aggregation ability to *E. coli* F18+ of *L. reuteri* decreased significantly after LiCl treatment compared to the untreated strain (*p* < 0.05), while *L. plantarum* showed that LiCl treatment did not impair its co-aggregation ability to *E. coli* (Figure 3).

### 3.2. Determination of Bacterial Hydrophobicity

In particular, *L. plantarum* and *L. reuteri* showed a significant decrease in bacteria in the aqueous phase after a chloroform treatment (82.83 ± 1.29% for untreated and 49.65 ± 1.45% for chloroform treated *L. plantarum*; 86.17 ± 2.12 for untreated and 55.22 ± 1.25% for chloroform treated *L. reuteri*; *p* < 0.0001) (Figure 3). The same drop was registered in the aqueous phase with toluene treatment for both bacterial strains (19.20 ± 1.69% for untreated and 6.91 ± 1.76% for toluene treated *L. plantarum*; 5.47 ± 1.28% for untreated and 1.94 ± 0.21% for toluene treated *L. reuteri*; *p* < 0.05).

### 3.3. Bacterial Adhesion Assay

**Figure 4:**
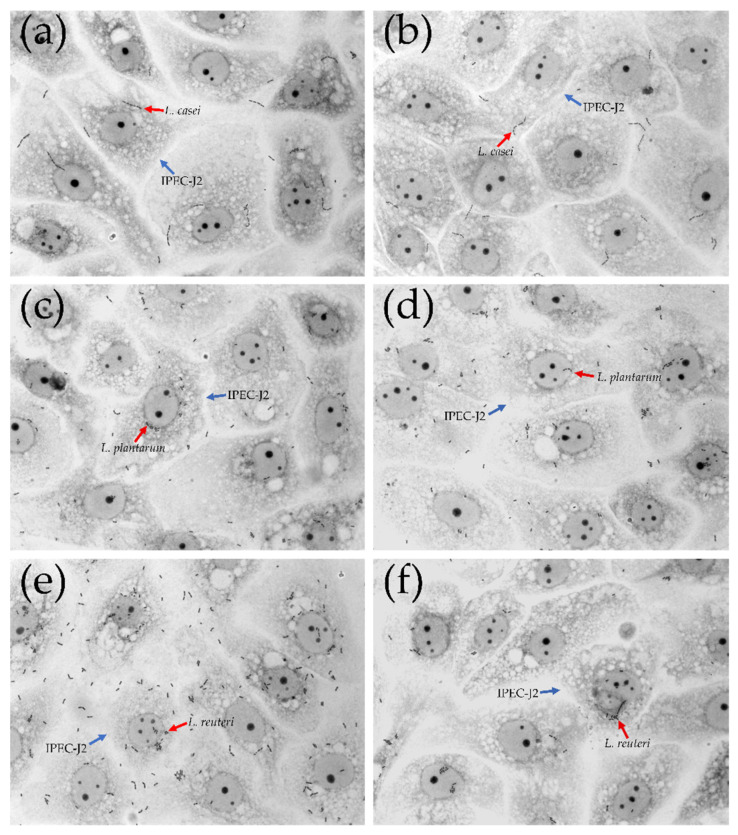
Adhesion of *L. casei*, *L. plantarum* and *L. reuteri* to IPEC-J2 cells. Representative microphotographs with 100× magnification (oil immersion). (**a**) *L. casei*; (**b**) *L. casei* + LiCl; (**c**) *L. plantarum*; (**d**) *L. plantarum* + LiCl; (**e**) *L. reuteri*; (**f**) *L. reuteri* + LiCl. Red arrows indicate lactobacilli strains; blue arrows indicate IPEC-J2 cells.

**Figure 5:**
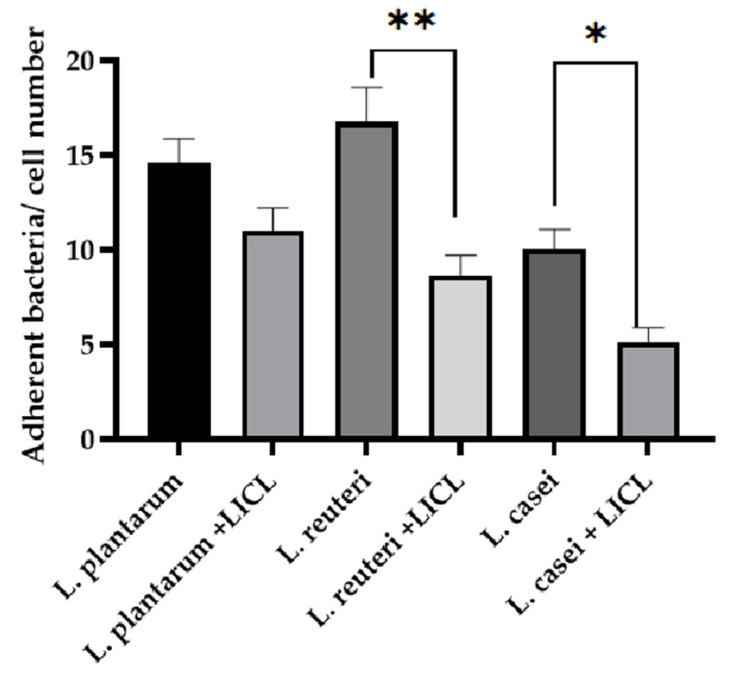
Count of adherent bacteria to IPEC-J2 cells, data are expressed as the mean value of adherent bacteria/cell number ± SEM of three independent experiments. * indicates a statistically significant difference (*p* ≤ 0.05); ** indicates a statistically significant difference (*p* ≤ 0.001).

*L. reuteri* showed the greatest adhesion capacity compared with *L. casei* and *L. plantarum* (Figure 4 and Figure 5). *L. plantarum* adhesion was not affected by the treatment with LiCl (14.64 ± 1.22 versus 11.04 ± 1.17). On the contrary, the treatment with LiCl significantly affected the adhesion capacity of *L. reuteri* and *L. casei (p* < 0.05). In particular, 16.77 ± 1.82 adherent bacteria/cell were counted in *L. reuteri*-treated cells while 8.63 ± 1.08 adherent bacteria/cell in *L. reuteri +* LiCl-treated cells. Regarding *L. casei*, 10.07 ± 1.02 adherent bacteria were counted in *L. casei*-treated cells whereas 5.14 ± 0.74 adherent bacteria in *L. casei* + LiCl-treated cells.

### 3.4. One and Two-Dimensional Gel Electrophoresis of Surface Proteins from Lactobacilli Strains

The extracted surface proteins resulted 1.478 µg/mL for *L. plantarum* and 0.970 µg/mL for *L. reuteri*, respectively.

**Figure 6:**
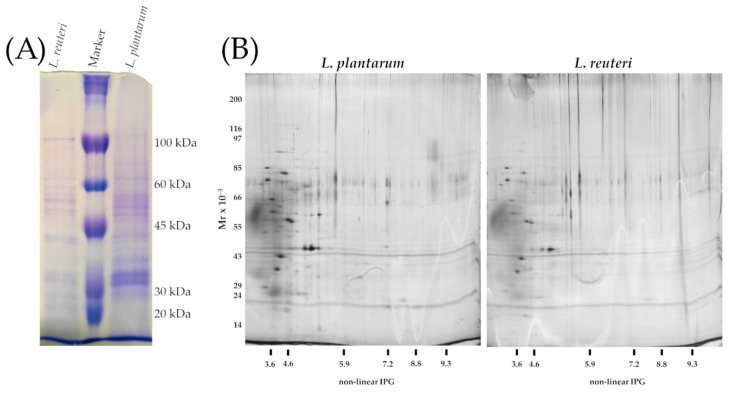
(**A**) SDS-PAGE of surface proteins. Equal amount of volume (14 μL) of the extracted polypeptides were loaded to each well; from left to right: S-layer extracted from *Lactobacillus reuteri*; Molecular weight marker (kDa), S-layer extracted from *Lactobacillus plantarum.* (**B**) Two-dimensional gel electrophoresis of S-layer polypeptides extracted from *L. plantarum* and *L. reuteri*.

One-dimensional denaturing gel revealed distinct protein bands, although quite qualitative differences in the intensity of bands of the surface proteins were observed (Figure S1). The observed polypeptides profile resulted in the range 20–100 kDa by comparison with the marker (Figure 6A). The two-dimensional gel electrophoresis did not show significant variations of protein spots between the two bacterial strains. The two-dimensional gel was mainly characterized by acid polypeptides ranging from 100 to 10 kDa for *L. plantarum* and *L. reuteri*, and no clusters were detected (Figure 6B).

## 4. Discussion

Alternatives to antibiotics are urgently needed due to the global concern regarding antibiotic resistance in livestock production systems [21,22,23].

Probiotics are defined as living microorganisms that can act in the treatment and prevention of infectious diseases when ingested in adequate amounts [24]. In particular, probiotic bacteria surface proteins have been demonstrated to intervene in cell protection and competitive adhesion against pathogens [9]. The presence of glyco-proteinaceous material at the bacterial cell surface is responsible of a higher hydrophobicity, whereas hydrophilic surfaces are associated with the presence of polysaccharides and affect the binding capabilities of probiotics [9]. In our study, a hydrophobic and basic phenotype of *L. plantarum* and *L. reuteri* was observed due to lactobacilli affinity to chloroform and ethyl acetate. In particular, *L. plantarum* showed higher hydrophobicity compared to *L. reuteri*, which may reflect their different colonizing ability. The values of MATS for toluene reflect the cell surface hydrophobicity or hydrophilicity capacities. Chloroform and ethyl acetate were used to assess their characteristics as electron donors (basic) and electron acceptors (acidic) of bacteria. In this study, the use of LiCl was adopted on probiotic cells to allow the selective and efficient removal of S-layer proteins. In particular, this step was carried out to evaluate the adhesive properties without surface proteins. Firstly, hydrophobic cell surfaces are reported for autoaggregation capacities that in some cases determine the intestinal colonization [25]. As expected, the treatment with LiCl strongly affected the hydrophobicity. The observed results suggest that *L. plantarum* and *L. reuteri* could potentially adhere to gut epithelium.

Other important characteristics of a probiotic bacteria are the auto- and co-aggregation capacity. The auto-aggregation is defined as the ability of cells of the same kind to self-adhere, and is recognized as an important predictive parameter for gut epithelium colonization as it allows bacteria to maintain a significant number of cells in the environmental niche [26]. Co-aggregation is defined as the binding of bacteria of diverse species (e.g., lactobacilli versus pathogens), and also evaluates the competitive inhibition capacity [26]. In fact, the formation of co-aggregates of lactobacilli with pathogens reduced pathogenic microorganisms’ ability to adhere to the intestinal epithelium [27]. In the present study, the auto-aggregation and co-aggregation to *E. coli* F18+ capacity of *L. plantarum* and *reuteri* indicate that cell surface proteins could be associated with lactobacilli aggregation properties and to specific binding capabilities in the gastrointestinal tract. Moreover, co-aggregation has been recognized as an important factor in the establishment and maintenance of a non-infectious gastrointestinal microflora. The observed co-aggregation with *E. coli* of *L. reuteri* and *L. plantarum* could suggest that lactobacilli constitute an important host-defence mechanism against infection. This hypothesis was also confirmed by the in vivo results described by Dell’Anno et al. [10], that registered a significative reduction in diarrhoea occurrence in weaned piglets’ diets supplemented with the investigated *L. plantarum* and *L. reuteri* strains. In this study, we focused on *Escherichia coli* F18+, a widely spread porcine enterotoxigenic pathogen, responsible for important economic losses in the pig industry [28,29]. *E. coli* F18+ strain has the ability to adhere to the intestinal surface by F18 adhesive fimbriae, and it is considered a major pathogen involved in the post-weaning disease and oedema disease [30]. Our study suggests that *L. plantarum* and *L. reuteri* can compete for adhesion with a pathogenic strain of *E. coli* through the formation of a barrier via auto-aggregation of by directly by co-aggregation with *E. coli*. It is recognized that auto-aggregation ability is related to adhesive properties of bacterial strains [31].

IPEC-J2 cell model is commonly used to investigate lactobacilli strains adhesive capacities [32]. The porcine intestinal epithelial IPEC-J2 cell line provides an excellent in vitro model for probiotic adhesion studies [33]. IPEC-J2 have been largely studied to assess probiotic adhesive properties and the anti-inflammatory activity of probiotic strains [34]. As shown by Tallon et al. [35], different chemical pre-treatments may influence the adhesive capacities of bacteria depending on the surface proteins that are involved in the adhesion process. In line with the study of Singh et al. [9,16], the pre-treatment with LiCl to remove cell surface proteins reduced the ability of *L. reuteri* to adhere to intestinal epithelium. On the contrary, *L. plantarum* was more resistant to LiCl treatment, probably due to the structural variations existing between the microorganism-associated molecular patterns which interact with the host pattern recognition receptors [36]. In this study, *L. reuteri* showed a higher adhesion ability compared to the behaviour of *L. casei* used as a control strain, in accordance with the study by Lähteinen et al. [37], which observed that *L. reuteri* isolates from the porcine intestine revealed the greatest adhesion capacity compared to other commensal lactobacilli in swine GIT (*L. amylovorus*, *L. mucosae* and *L. johnsonii*). In addition, specific strains of *L. reuteri* express different surface proteins capable of improving binding properties compared with a well-known probiotic such as *L. casei* [31,38,39,40]. Mucus binding abilities related to the production of mannose-sensitive adhesins have been reported for some *L. plantarum* and *L. reuteri* strains [25]. The observed adhesion properties could be due to the presence of mucus-binding proteins on cell surface of these two bacteria.

In line with other studies, the SDS-PAGE of cell surface proteins of *L. reuteri* and *L. plantarum* revealed the presence of several bands of varying length if compared with the protein fragments of the marker in the range from 100 to 20 kDa [9,41]. Two-dimensional gel electrophoresis confirmed the acidic prevalence of bacterial surface proteins that are typical subunits of surface proteins of lactobacilli [42]. In visual evaluation, the polypeptide profile was very similar, and the molecular range confirmed the previously observed results of one-dimensional electrophoresis (SDS-PAGE). In line with our results, Wang et al. [41] observed that a protein of approximately 37 kDa for *L. plantarum* strain. This protein, the glyceraldehyde-3phospated dehydrogenase (GAPDH), plays an important role for the adhesion properties of *L. plantarum* [41]. It has been shown that CscA, CscB and CscD proteins of *L. plantarum* are functionally related to a cell-surface protein complex that could play a role in sugar acquisition. In particular, a cscB gene product known as co-aggregation-promoting factor (Cpf) could be removed from *L. plantarum* surface by treating bacterial cells with LiCl (5M) and reattached by salt removal, restoring its co-aggregation ability [43]. Mannose-binding proteins of *L. plantarum* WCFS1 are similar to a mucus-binding protein from *L. reuteri* that are likely to be involved in the interaction with the host [44]. *L. reuteri* 100-23 possesses a high-molecular-mass surface protein (Lsp) and methionine sulfoxide reductase B (MsrB), which both contribute to adherence in the gut [45]. *L. reuteri* JCM1081′s adhesive property appeared to be mediated by the presence of a surface protein of approximately 29 kDa with an important similarity to the putative ATP-binding cassette transported protein CnBP [46]. In our study, *L. plantarum* and *L. reuteri* revealed a quite similar surface profile, with polypeptides ranging from 20 to 100 kDa; however, their characterization and functions need to be confirmed by further experiments.

The survival of probiotics during gastrointestinal transit and their adhesion on the intestinal surface are important prerequisites for the colonization and competitive inhibition proprieties. The strains here analysed have been previously demonstrated to greatly survive the gastro-intestinal conditions [10], which is an important prerequisite in probiotic efficacy. The results here presented clearly indicate the relation between cell surface characteristics and adhesion ability of *L. reuteri* and *L. plantarum.* Therefore, more studies are needed to identify the particular proteins involved in these mechanisms, in order to select the most suitable probiotic strains for application in weaning piglets. The findings of the current study can be extrapolated to those situations in which it could be relevant to formulate a probiotic product to target performance or health-related challenges in the pig’s life.

## 5. Conclusions

Our study showed that LiCl significantly inhibited the autoaggregation and co-aggregation to *E. coli* of *L. plantarum* and *L. reuteri*. In addition, a strong affinity to chloroform indicated the hydrophobic and basic phenotypes of these lactobacilli. The adhesive capacity of *L. reuteri* on IPEC-J2 cells was significantly reduced after a pre-treatment with LiCl, suggesting a pivotal role of surface proteins in the epithelial adhesion. *L. plantarum* and *L. reuteri* revealed an important influence of the adhesive proteins related to their probiotic characteristics, further research is necessary to better address the mechanisms of these proteins in the gut colonization, and also in relation to intestinal microbiota. Additionally, *L. plantarum* and *L. reuteri* might be suitable candidates for further study, due to their protective effects against *E. coli* infections in weaned piglets.

## Data Availability

The data presented in this study are available within the article.

## Supplementary Materials

The following are available online at https://www.mdpi.com/article/10.3390/microorganisms9081587/s1.
