# Supplementary material for: Evaluation of Adhesive Characteristics of L. plantarum and L. reuteri Isolated from Weaned Piglets
Source: Microorganisms. 2021 Jul 26;9(8):1587. doi: 10.3390/microorganisms9081587 (PMC8400209; doi:10.3390/microorganisms9081587)
Supplement: Supplementary file 1 [file microorganisms-09-01587-s001.zip › microorganisms-1273379-supplementary.pdf]

Supplementary Materials

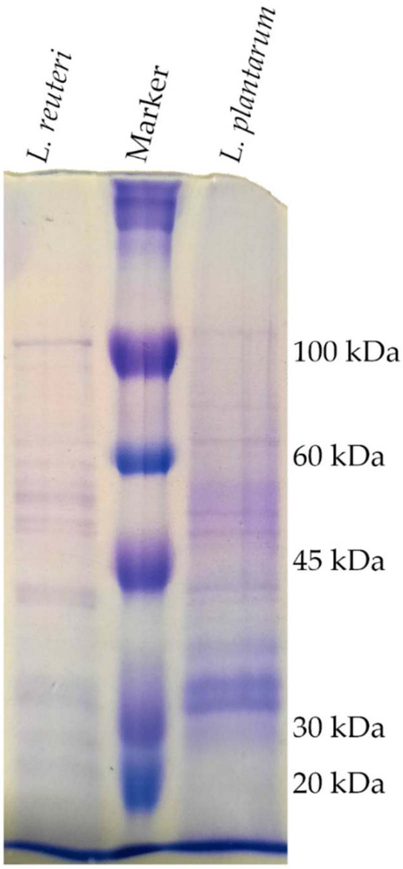

Figure S1. SDS-PAGE of surface proteins. Equal amount of volume (14 uL) of the extracted polypeptides were loaded to each well; from left to right: S-Layer extracted from *Lactobacillus reuteri*; Molecular weight marker (kDa), S-Layer extracted from *Lactobacillus plantarum.*
